# Guiding significance of intraoperative frozen section for range of judging incisal edge of Esophageal Carcinoma

**DOI:** 10.12669/pjms.37.5.3910

**Published:** 2021

**Authors:** Fei He, Chen Wang, Weichao Liu, Gongning Shi

**Affiliations:** 1 Fei He, Department of Thoracic and Cardiovascular Surgery, The Second Xiangya Hospital of Central South University, Changsha 410011, Hunan, China. Department of Thoracic and Cardiovascular Surgery, The Huaihe Hospital of Henan University, Kaifeng 475300, Henan, China; 2 Chen Wang, Department of Thoracic and Cardiovascular Surgery, The Huaihe Hospital of Henan University, Kaifeng 475300, Henan, China; 3 Weichao Liu, Department of Thoracic and Cardiovascular Surgery, The Huaihe Hospital of Henan University, Kaifeng 475300, Henan, China; 4 Gongning Shi Department of Thoracic and Cardiovascular Surgery, The Huaihe Hospital of Henan University, Kaifeng 475300, Henan, China

**Keywords:** Cancerous residue of incisal edge, Esophageal carcinoma, Intraoperative frozen section

## Abstract

**Objective:**

To explore guiding significance of intraoperative frozen section for judging incisal edge range of esophageal carcinoma.

**Methods:**

A retrospective descriptive research design was used to collect the clinical and pathological data of 205 patients with esophageal cancer who were treated in Huaihe Hospital of Henan University from March 2012 to July 2015. Among them, 46 patients’ esophageal margins were made into intraoperative frozen sections.

**Results:**

In the 205 cases, nine cases were diagnoses with upper incisal edge cancerization, accounting for 4.39%, and five cases were diagnosed with lower incisal edge cancerization, accounting for 2.4%. There were 14 cases in total, accounting for 6.83%. four cases showed positive residual end of intraoperative frozen section.

**Conclusion:**

The cancerous focus residue of incisal edge in esophageal carcinoma is not uncommon. Intraoperative frozen section is helpful to judge the proper excision length of esophageal carcinoma.

## INTRODUCTION

In the past few years, the incidence of esophageal adenocarcinoma has gradually increased. At present, deaths from esophageal cancer account for one-sixth of cancer-related deaths.^1^ Esophageal cancer has high morbidity and mortality, early symptoms, early hematology and lymph node spread. therefore, radical surgery is still the only treatment of choice.^2^,^3^ It has been reported that the depth of tumor invasion, lymph node involvement, and involvement of the distal and proximal resection margins have been shown to be associated with poor prognosis in patients undergoing resection.^4^-^6^ Another investigator reported that involvement of the circumferential margin in patients with esophageal cancer is associated with increased risk of local recurrence and decreased long-term survival.^7^ Therefore, in patients with esophageal cancer, it is very important to choose the appropriate range of esophagus resection. Studies have reported that the application of intraoperative frozen section in breast cancer, ovarian tumor and other operations has important guiding significance for the safety of surgery and the effectiveness of treatment.^8^,^9^ Shiraki T et al. also reported the importance of intraoperative frozen section in judging the margin of extrahepatic cholangiocarcinoma.^10^

However, there are few reports on the use of intraoperative frozen section to determine the margin of esophageal cancer. The pathological characteristics of 205 patients with esophageal cancer who underwent surgical treatment in Huaihe Hospital of Henan University were analyzed in this study to explore the guiding significance of intraoperative frozen section in judging the margin of esophageal cancer. The specific situation is reported as follows.

## METHODS

The pathological characteristics of 205 cases with esophageal carcinoma receiving operative treatment at The Huaihe Hospital of Henan University from March 2012 to July 2015 were collected. General clinical characteristics of these patients, incisal edge cancerization of esophageal carcinoma focus and the results of frozen section of 46 cases were analyzed and discussed. Among the 205 patients, the number of male patients was higher than that of female patients, with the ratio of 2.1:1. The age of patients ranged from 38 to 82, with the average age of 62 and median age of 62. There was only one case below the age of 40 (0.49%), 8 cases at the age of 40~49 (3.90%), 58 cases at the age of 50~59 (28.29%), 90 cases at the age of 60~69 (43.90%), 39 cases at the age of 70~79 (19.02%) and three cases above the age of 80 (1.46%). Histopathologic type was mainly squamous carcinoma, accounting for 94.1%. Pathomorphological type was mostly ulcerative type, accounting for 67.2%. The positive rate of lymphatic metastasis was high, 43.4%. Among the 205 cases, multi-stage cancerization happened to four cases, accounting for 1.95%.

### Ethical approval

The study was approved by the Institutional Ethics Committee of the Huaihe Hospital of Henan University, dated February 25, 2020 and written informed consent was obtained from all participants.

The incisal edge tissues in the anastomat were sent for inspection. The incisal edges were directly put on the tissue tray, embedded by the embedding medium, placed on the freezing bench, frozen for one to two minutes at the constant temperature of 25^0^C and then cut into slices with the thickness of 5µm. Finally, the slices were directly pasted on the clean glass slide, dried with cold air and stained by conventional HE. According to the frozen section results, if there is no carcinoma residue on the upper and lower incisal edges, suturing would be performed according to the original plan; if there is no carcinoma residue on the incisal edge, the resection would be extended again. And, the incisal edge would be sent for intraoperative frozen section examination again until there is no carcinoma residue on the incisal edge. Conventional pathological examination of paraffin section was conducted for the remaining tissues of intraoperative frozen section so as to contrast with the freezing result.

## RESULTS

Incisal edge cancerization results of 205 cases with esophageal carcinoma are shown in Table-I. The number of cases with upper incisal edge cancerization accounted for 4.39%, while the number of cases with lower incisal edge cancerization accounted for 2.4%. There were total 14 cases (6.83%). Among the 205 cases, esophageal incisal edges of 46 cases were made into intraoperative frozen sections, including four cases with positive result (8.7%) and 42 cases with negative result (91.3%). The results of intraoperative frozen section confirmed to postoperative conventional pathological results.

**Table-I:** Positive rate of incisal edges of 205 cases with esophageal carcinoma esophageal carcinoma.

Group	Positive	Negative	Total No.	Positive rate (%)
Frozen section group	4	42	46	4/46 (8.70%)
Non-frozen section group	14	145	159	14/159 (8.80%)

Pathological characteristics of 14 cases with positive incisal edge are shown in Table-II. Positive incisal edges mainly appeared to the upper incisal edges, and the ratio of men to women was 3.7:1. Histological type was mainly squamous carcinoma, and morphological type was mainly ulcerative type. The positive rate of lymphatic metastasis was 64.3%. The positive rate of esophageal carcinoma with incisal edge <3cm away from the tumor was higher than that of the esophageal carcinoma with incisal edge ≥ 3cm away from the tumor, indicating that the farther the incisal edge is away from the tumor, the lower the positive rate of incisal edge. The positive rate of residual end of esophageal carcinoma with deep infiltration degree was high.

**Table-II:** Pathological characteristics of 14 cases with positive incisal edge theological results.

Item		No.	%
Upper/lower			
	Upper	9	64.3
	Lower	5	35.7
Gender			
	Male	11	78.6
	Female	3	21.4
Age			
	≤40	1	7.1
	>40~≤70	13	92.9
Histological classification			
	Squamous carcinoma	13	92.9
	Adenocarcinoma	1	7.1
Morphological classification			
	Ulcerative type	9	64.3
	Medullary type	4	28.6
	Constrictive type	1	7.1
Lymphatic metastasis			
	Positive	9	64.3
	Negative	5	35.7
Distance with incisal edge			
	<3cm	10	71.4
	≥3cm	4	28.6
Depth of infiltration			
	Deep muscularis	2	14.3
	The whole layer reaches the outer membrane	12	85.7

## DISCUSSION

At present, the positive incisal edge problem in esophageal carcinoma fails to receive much attention. For the operations of esophageal carcinoma and gastric cancer, anastomotic fistula and anastomotic stenosis are the most important complications. For a long time, clinicians have attached great importance to prevention and treatment of anastomotic fistula and anastomotic stenosis. Many researches have been conducted clinically, and many articles have been published. According to reports^11^, the incidence of esophagogastric anastomotic leakage in our country is 3-25%. The positive margin of the esophagus should also cause equally important attention. Wu J et al.^12^ have proved that positive circumferential resection margin is associated with poor prognosis in patients with esophageal cancer, particularly in patients with T3 stage disease and patients receiving neoadjuvant therapy. And preoperative circumferential resection margin prediction has proved an effective strategy in tailoring neoadjuvant and surgical strategies in rectal cancer, reducing rates of margin positivity and locoregional recurrence.^13^ This approach has not yet been explored in oesophageal adenocarcinoma. In this study, the occurrence rate of incisal edge carcinoma was 6.83%. Positive incisal edge in esophageal carcinoma makes radical operation for esophageal carcinoma become palliative surgery. Generally, the relapse can be seen visually after 2-4 months, and the effects of reoperation and chemoradiotherapy are poor. Thus, there is large number of complications of the clinical treatment. Postoperative cancerous focus residue directly influences patients’ survival time, and it is an independent risk factor influencing postoperative relapse of esophageal carcinoma.^14^,^15^ However, this problem is rarely focused, and there are few relevant articles without enough attention.

Esophageal carcinoma surgery should follow the principle of maximum excision of tumor tissues and maximum reservation of normal tissues. Inberg MV et al.^16^ reported that the distance between near-end incisal edge and the upper tumor edge should not be less than 5cm for the proper excision length for esophageal carcinoma surgery, and the esophagus should be excised completely at the far end. Huang KC et al.^17^ has been reported that subtotal esophagectomy and cervical esophagogastrostomy should be performed to reduce relapse of residual esophageal carcinoma at the esophageal broken end and long-term residual esophagus. Wang J et al.^18^ has found that positive residual end may farthest happen at 8cm of upper carcinoma excision and 4cm of lower carcinoma excision. And they believed that the positive rate of upper residual end with the excision extension>0.5cm does not decline with the increase of excision length range. At present, NCCN diagnosis and treatment guideline suggests that the distance between esophageal incisal edge and upper tumor edge should be greater than 5cm. However, the positive rate of esophageal residual end is still high. JGCA guidelines recommend that in patients with gastric cancer, a distance of at least 2 cm between the tumor and the resection line should be maintained to avoid the risk of marginal invasion.^19^ Most of the scholars have suggested increase excision length of esophagus can lower the positive rate of residual end of esophageal carcinoma. The excessive excision length will increase the operation more complicated and will create more complications, and will effect on quality of life. Thus, we recommend intraoperative frozen section to determine the excision length of esophagus. And our study found that among the 14 patients with positive pathological results of the esophageal resection margin, the proportion of those with a resection length of less than 3 cm (71.4%) was higher than that of those with a resection length of ≥ 3 cm (28.6%).

### Reasons for positive incisal edge of esophageal carcinoma and gastric cancer

After the resection of esophageal carcinoma, the common reasons for carcinoma residual at the broken end of esophagus are as below:

1. Main carcinoma residue, which is caused by insufficient excision length.
2. Peritumor microsatellite lesions transferring through lymphatic vessel.
3. Esophageal carcinoma has the characteristics of multicenter origins, in-wall infiltration and jumping metastasis.

The latter two situations have little relation with excision length, and cannot be confirmed by observation and touch. Thus, the existing operation methods are difficult to completely avoid postoperative incisal edge carcinoma residue.^20^

### Judgement of intraoperative incisal edge carcinoma of esophageal carcinoma

Some hospitals apply Lugol’s solution to examine intraoperative incisal edge or esophageal mucosal lesions in esophageal carcinoma, but such method can only hint esophageal mucosa lesion and cannot determine the nature. Besides, there are certain false positive and false negative proportions. We use the anastomat to anastomose esophagus and stomach in the operation, and the incisal edge in the anastomat was sent to make frozen sections. Intraoperative frozen section can provide histopathological evidence, accurately judge incisal edge cancerization, and guide esophagus excision length. In addition, it takes a little time, without impacting the operation process. In this study, intraoperative frozen section was made for 46 cases, including four cases with positive result. Thus, esophagus excision length was increased to reduce postoperative relapse risk. Thus, the authors consider that the incisal edge of esophageal carcinoma should be frozen rapidly to determine the excision length of esophagus.

### Limitations of this study:

1. For patients with positive residual cancer at the resection margin, whether they need to undergo pathological examination again after resection requires further discussion and research in the future.
2. The number of cases included in this study is not enough, and it is necessary for future studies to further expand the sample size and draw more convincing conclusions.

## CONCLUSION

In general, our research has proved that the longer the distance between the resection margins of esophageal cancer, the lower the positive rate of resection margins and the deeper the tumor invasion, the higher the positive rate of resection margins. Therefore, intraoperative frozen section is of great significance in guiding the scope of surgical resection of esophageal cancer.

### Authors’ Contributions:

**FH and GS** designed this study and prepared this manuscript, and are responsible and accountable for the accuracy or integrity of the work.

**WL** collected and analyzed clinical data; **CW** significantly revised this manuscript.
